# Composite non-clinical interventions for a safe cesarean section rate reduction: results of a pre-post interventional study

**DOI:** 10.1186/s12884-021-04245-y

**Published:** 2021-11-19

**Authors:** A. Fruscalzo, K. Reinecke, A. P. Londero, M. Gantert

**Affiliations:** 1 Obstetrics and Gynecology, St. Franziskus Hospital of Ahlen, Ahlen, Germany; 2 Present address: Obstetrics and Gynecology, University Hospital of Fribourg, Fribourg, Switzerland; 3 Obstetrics and Gynecology, University Hospital of Udine, Udine, Italy; 4 Ennergi Research (non-profit organisation), Lestizza, UD 33050 Italy

**Keywords:** Intervention, Cesarean section reduction, Vaginal delivery, Operative delivery, Delivery complications

## Abstract

**Objective:**

To evaluate the impact on cesarean section (CS) rate with of a program of multiple non-clinical interventions targeted at health-care professional within a hospital maternity ward.

**Materials and methods:**

Retrospective quasi-experimental pre-post intervention study with an historical control group conducted in a second-level teaching hospital. All women who gave birth in the period 2014 to 2018 were included. A series of multiple non-clinical interventions including a dedicated team of obstetricians for delivery room and antenatal counseling, monthly internal audits and physician education by local opinion leader were prospectively introduced from September 2016. The primary outcome of the study was the CS rate. The incidences of operative vaginal delivery, 3rd−/4th-degree perineal tears and further maternal and neonatal complications were considered as secondary outcomes.

**Results:**

The CS rate dropped from 33.05 to 26.06% after starting the interventions (*p* < 0.01); in particular, the cumulative rate of CS performed during labor decreased significantly from 19.46 to 14.11% (*p* < 0.01). CS reduction was still statistically significant after multivariate correction (OR = 0.66, CI.95 = 0.57–0.76, *p* < 0.01). Results further showed an increased prevalence of 3rd-degree perineal tears (0.97% versus 2.24%, *p* < 0.01), present also after correcting for possible confounding factors (OR = 2.36, CI.95 = 1.48–3.76, *p* < 0.01). No differences were found in the rate of vaginal-operative births and further maternal complications, while the composite neonatal outcome was found to be improved (OR = 0.73, CI.95 = 0.57–0.93, *p* = 0.010).

**Conclusions:**

The introduction of multiple non-clinical interventions can significantly reduce the CS rate. However, beside an improvement in neonatal composite outcome, a potential increase in high-degree perineal tears should be taken in account.

**Supplementary Information:**

The online version contains supplementary material available at 10.1186/s12884-021-04245-y.

## Introduction

Rising concerns have been expressed due to the continuous increase in cesarean section (CS) rate worldwide [1]. Epidemiological data describe an extreme variability in its geographical distribution, reflecting the effect played by peculiar differences in the socio-cultural background and in the organization of local medical care systems [2]. According to WHO recommendations, a cesarean delivery rate > 10–15% is associated with additional risks and higher healthcare costs without further advantages for mother and newborn [3]. Furthermore, the management and counselling concerning short and long-term maternal and neonatal outcomes have become a main topic for obstetricians [4].

In line with this evidence, health care systems focus on cesarean delivery rate as one of the most important criteria of medical quality in obstetrics [5]. Strategies focused on the reduction of cesarean delivery rate have been supported by several international gynecological and obstetrics societies [6–8]. As several reasons have been advocated for the increasing CS rate, solutions appear to be overly complex and challenging for medical institutions and gynecologists. However, evidence shows that a reduction of the cesarean delivery rate by favoring practices supporting natural birth is not only advisable, but also feasible [9, 10]. According to the WHO recommendations, non-clinical interventions, i.e. interventions not linked to patient’s clinical conditions, seems to be those ones easier to put into practice. They could be subdivided, according to their target, in addressed at women, at health-care institutions facilities and systems, or health-care professionals [11]. The objective of the study was to evaluate the impact of a program of interventions targeted at obstetrics health-care professionals aimed at supporting vaginal birth and safely reducing CS rate. The incidences of operative vaginal delivery and of maternal and neonatal complications were also evaluated.

## M&M

### Study design and setting

This was a retrospective quasi-experimental pre-post intervention study with an historical control group conducted in a second level German university teaching hospital. A non-clinical intervention addressed to different target stakeholders was started in September 2016. Outcome measures were evaluated from this time to December 2018 and compared to an historical cohort from January 2014 to August 2016 [12]. All patients delivering at our institution during the study-period were included for evaluation of delivery outcome, without any exclusion criteria. All methods were carried out in accordance with relevant guidelines and regulations under Ethics approval. The local institutional review board approved the study (Klinisches Ethikkomitee - St. Franziskus-Hospital Ahlen, Sitzungsprotokoll SFA-OrgaHB-8.-FB 1). An informed consent was not required because of the retrospective nature of the analysis. Patient’s data were analyzed in anonymous form.

The start of interventions was preceded by the set-up of a dedicated task force in charge of carrying out an analysis of current statistical landmarks and make focused proposals to support the practice of natural childbirth and safely reduce the CS rate. The group was composed of all professional figures involved in the process of childbirth, including resident and consultant obstetricians and midwives. In an as-is analysis, the previous year’s obstetric annual data on pregnancy and delivery outcomes of our hospital (2015) were examined and compared with the data from all over the region and the whole country (data not shown). Proposals for interventions were selected using a Delphi strategy [13]. After discussion among the respective groups of professionals, a final project was drawn up and presented in a dedicated meeting to all clinic employees.

### Proposed interventions

The interventions proposed by the working group were subdivided according to the target addressed, both oriented at the hospital’s organizational structure and maternity health-care professional’s skills.

Concerning the local hospital organization level, one intervention indicated was the definition of a team of gynecologists responsible for the delivery room and, in general, for the accomplishment of the study. To guarantee proper continuity, two senior gynecologists, one of them (AF) having greater experience and a German sub-speciality in perinatal care, and three further resident gynecologists were included in this team. One of the resident gynecologists and a senior gynecologist were defined as primarily responsible for the delivery room during the regular working hours, while the others were in a back-up position as needed. These medics periodically changed their roles, respectively every 6 months the resident gynecologists and during the last year, the senior doctor. The task of this team was to be responsible for the direction of the delivery room during regular working hours. This included two daily visits to the delivery room, one in the early morning and the second in the early evening, respectively at the beginning and at the end of the regular working day, as well as for management of the pregnant women presenting at the delivery room for deliveries and any type of emergencies. Daily visits to the delivery room were performed to assess the current status of the pregnant women admitted for delivery and discuss the further management together with the midwifery team. A second intervention was undertaken to better counsel pregnant women about delivery mode and select those without contraindications for vaginal delivery or at risk of major complications. The prenatal interview, already offered to all patients planning to deliver in our hospital between the 34th and the 36th week of gestation, would only be performed by the two senior gynecologists dedicated to the delivery room.

The following other interventions were also started, to implement the theoretical and practical competence of health-care personnel. They included monthly obstetrical morbidity-&-mortality conferences carried out under the responsibility of the resident gynecologist under the supervision of one of the two senior gynecologist. The midwifery team participated in each session of the educational program. A one-to-one practical educational training of the resident gynecologist was also provided by the senior gynecologist responsible for the delivery room as part of this intervention.

### Main and secondary outcomes

The main outcome of the study was the overall rate of CS. A sub-analysis by Robson groups was also undertaken [14]. Operative vaginal delivery, as well as the incidence of major maternal and neonatal complications were evaluated as secondary outcomes. Maternal complications included: Peripartal mortality, blood loss > 1000 ml, hematomas, hysterectomy, fever, pulmonary embolism and deep vein thrombosis or lung embolism, 3rd-or 4th-degree perineal lacerations. Neonatal complications included the following concerns: Perinatal mortality, 5th minute Apgar < 7, arterial pH < 7.10, need for mask ventilation or neonatal intubation and postnatal admission at the neonatal intensive care unit (NICU). Also, according to the diagnosis on admission, NICU hospitalization was considered as childbirth related in the case of respiratory distress due to poor APGAR score or acidotic umbilical artery pH or neonatal infection, but not due to hyperbilirubinemia or hypoglycemia, and prematurity. A negative maternal composite outcome was also evaluated as follows: Perineal tears of 3rd- or 4th-degree, post-partum hemorrhage, hysterectomy, fever, pulmonary embolism, hematoma, and post-operative complications. A negative composite neonatal outcome was evaluated as follows: Childbirth related NICU hospitalization, Apgar score at 5th minute < 7, cord blood pH < 7.1, cord blood base-excess <− 12 and neonatal tracheal intubation. Data were achieved both through the internal medical charts and the annual records provided by the Quality Assurance Office of North Rhine-Westphalia (Agency for Quality Assurance Nordrhein-Westfalen, QS-NRW).

### Statistical analysis

The analysis was performed with R software (R: A Language and Environment for Statistical Computing, Vienna, Austria 2020 – version 3.6.3). The population was divided into two groups according to the period before (group 1) and after (group 2) the introduction of the intervention. The target sample size was estimated using a calculation based on the ability to detect 5% changes in CS prevalence between groups and to an expected 600 deliveries per year [9]. Thus, considering a significance level of 0.05, and 80% power, it was estimated the required minimum sample size to be 1297 patients per group that requires the inclusion of at least a two-year period per group. The normality of the sample distribution was assessed using the Kolmogorov Smirnov test. Data were presented as mean and standard deviation (±SD) or median and interquartile range (IQR) in case of continuous variables and as a percentage and absolute values in case of discrete variables. Also, the odds ratio (OR) with the relative 95% confidence interval (95% CI) were presented. To compare the groups, as appropriate, the following tests were used: t-test, Wilcoxon test, chi-square test, or Fisher’s exact test. Finally, univariate and multivariate logistic regression analyses were performed. As dependent variables the main outcomes were selected: CS occurrence, maternal composite outcomes, fetal composite outcomes. While the independent variables included were all the possible confounding factors. The initial multivariate models included the interaction terms that were excluded from the final model if not statistically significant. The effect of the intervention on CS rate was also assessed by interrupted time series analysis. The time series of cesarean section rates were divided into two segments (the first segment, before intervention started, and the second, after intervention started). Hence, the segmented regression was applied to measure immediate changes in CS rate and changes in its trend. The results were presented as regression coefficients with a 95% CI.

## Results

### Population description

During the study period, which lasted 5 years, 4997 women gave birth in our center (2672 before the intervention and 2325 after the intervention). Table 1 shows pregnancy characteristics and outcomes in both the pre- and post-intervention periods. No significant differences were found in maternal and pregnancy characteristics. Table 2A shows the distribution of pregnancies among the Robson classes before and after the implementation of the intervention. The only difference detected was a nonsignificant decrease after intervention in the rate of labor induction among multiparous single term cephalic pregnancies without a history of previous CS (Robson 4a). Neonatal weight and fetal presentation at delivery were not statistically different before and after the intervention (Table 3).

**Table 1:** Characteristics of population and maternal outcomes

	Group 1 (2672)	Group 2 (2325)	*p*
**Maternal and pregnancy characteristics**			
Age (years)	30.05 (±5.21)	30.12 (±5.15)	0.604
Pre-pregnancy BMI (kg/m^2^)	25.71 (±5.71)	25.90 (±5.68)	0.240
Gestational age at delivery (weeks)	39.29 (±1.68)	39.25 (±1.69)	0.403
Gravidity	1.00 (0.00–2.00)	1.00 (0.00–2.00)	0.509
Parity	1.00 (0.00–1.00)	1.00 (0.00–1.00)	0.376
Twins	1.57% (42/2672)	1.46% (34/2325)	0.752
Previous CS	16.65% (445/2672)	17.38% (404/2325)	0.498
Pre-pregnancy DM	1.24% (33/2672)	0.99% (23/2325)	0.410
GDM	7.41% (198/2672)	7.70% (179/2325)	0.700
PRHDs	4.83% (129/2672)	4.43% (103/2325)	0.505
Placenta previa	0.60% (16/2672)	0.43% (10/2325)	0.408
**Labor characteristics and maternal outcomes**			
Labor induction	25.49% (681/2672)	25.46% (592/2325)	0.984
Labor augmentation	21.26% (568/2672)	22.19% (516/2325)	0.423
Mode of delivery			
Vaginal delivery	57.37% (1533/2672)	65.59% (1525/2325)	< 0.01
Operative vaginal delivery	9.58% (256/2672)	8.34% (194/2325)	0.128
Cesarean section	33.05% (883/2672)	26.06% (606/2325)	< 0.01
Primary CS	13.59% (363/2672)	11.96% (278/2325)	0.086
Secondary CS	19.46% (520/2672)	14.11% (328/2325)	< 0.01
Emergency CS	1.76% (47/2672)	1.42% (33/2325)	0.340
Duration of CS	40.35 (±14.31)	40.11 (±12.71)	0.737
Hospitalization in CS subgroup (days)	5.22 (±2.49)	4.82 (±2.13)	< 0.01
Hospitalization in all (days)	3.98 (±2.18)	3.57 (±1.86)	< 0.01
Episiotomy	20.13% (538/2672)	16.09% (374/2325)	< 0.01
Perineal tears			
1st-degree	11.26% (301/2672)	12.90% (300/2325)	0.076
2nd-degree	11.86% (317/2672)	13.42% (312/2325)	0.098
3rd-degree	0.97% (26/2672)	2.24% (52/2325)	< 0.01
4th-degree	0.11% (3/2672)	0.04% (1/2325)	0.628
PPH	2.10% (56/2672)	1.38% (32/2325)	0.054
Hysterectomy	0.11% (3/2672)	0.04% (1/2325)	0.388
Fever	0.11% (3/2672)	0.09% (2/2325)	0.770
Pulmonary embolism	0.07% (2/2672)	0.04% (1/2325)	0.647
Hematoma	0.45% (12/2672)	0.34% (8/2325)	0.558
Post-operative complications	0.82% (22/2672)	0.69% (16/2325)	0.583
Other complications	0.34% (9/2672)	0.34% (8/2325)	0.965
Maternal composite outcome ^a^	3.63% (97/2672)	4.04% (94/2325)	0.448

*BMI* body mass index, *CS* cesarean section, *DM* diabetes mellitus, *GDM* gestational diabetes mellitus, *PRHDs* pregnancy related hypertensive disorders, *PPH* post-partum hemorrhage

^a^Maternal composite outcome consisting of one or more of the following outcomes: Perineal tears of 3rd- or 4th-degree; post-partum hemorrhage; hysterectomy; fever; pulmonary embolism; hematoma; post-operative complications

**Table 2:** Distribution of cases among Robson classes (A) and prevalence of CS stratified for Robson classes (B)

**A)**	**Group 1 (2672)**	**Group 2 (2325)**		***p***
**Robson classes prevalence**				
1	23.02% (615/2672)	22.54% (524/2325)		0.687
2a	11.64% (311/2672)	12.60% (293/2325)		0.298
2b	1.80% (48/2672)	1.51% (35/2325)		0.422
3	27.32% (730/2672)	27.10% (630/2325)		0.859
4a	9.47% (253/2672)	8.13% (189/2325)		0.096
4b	0.94% (25/2672)	0.60% (14/2325)		0.181
5a	12.39% (331/2672)	12.95% (301/2325)		0.554
5b	1.98% (53/2672)	2.41% (56/2325)		0.305
6	2.25% (60/2672)	2.19% (51/2325)		0.901
7	1.01% (27/2672)	1.46% (34/2325)		0.147
8	1.57% (42/2672)	1.46% (34/2325)		0.752
9	0.60% (16/2672)	0.30% (7/2325)		0.121
10	6.03% (161/2672)	6.75% (157/2325)		0.294
**B)**	**CS in group 1**	**CS in group 2**	**Difference (%)**	***p***
**Robson classes: CS prevalence**				
1	26.67% (164/615)	16.22% (85/524)	−10.45	< 0.01
2a	39.23% (122/311)	29.01% (85/293)	−9.22	< 0.01
2b	100.00% (48/48)	100.00% (35/35)	0	1.000
3	3.70% (27/730)	2.38% (15/630)	−1.32	0.161
4a	12.65% (32/253)	3.70% (7/189)	−8.86	< 0.01
4b	100.00% (25/25)	100.00% (14/14)	0	1.000
5a	64.05% (212/331)	51.50% (155/301)	−12.55	< 0.01
5b	100.00% (53/53)	96.43% (54/56)	−3.57	0.165
6	98.33% (59/60)	96.08% (49/51)	−2.25	0.465
7	96.30% (26/27)	85.29% (29/34)	−11.01	0.152
8	71.43% (30/42)	47.06% (16/34)	−24.37	0.031
9	100.00% (16/16)	100.00% (7/7)	0	1.000
10	42.86% (69/161)	35.03% (55/157)	−7.83	0.153

**Table 3:** Neonatal characteristics and outcomes

	Group 1 (2714)	Group 2 (2359)	*p*
Neonatal male sex	50.99% (1384/2714)	51.80% (1222/2359)	0.566
Fetal presentation at delivery			
Cephalic presentation (LOA or ROA)	91.97% (2496/2714)	92.92% (2192/2359)	0.201
Other cephalic presentation	3.17% (86/2714)	2.33% (55/2359)	0.070
Breech presentation	3.98% (108/2714)	4.28% (101/2359)	0.589
Transverse/shoulder presentation	0.88% (24/2714)	0.47% (11/2359)	0.073
Neonatal weight (grams)	3390.55 (±533.33)	3404.58 (±546.74)	0.357
Apgar score 1st minute < 7	6.23% (169/2714)	5.34% (126/2358)	0.180
Apgar score 5th minute < 7	2.17% (59/2714)	1.36% (32/2358)	0.029
Apgar score 10th minute < 7	0.29% (8/2714)	0.17% (4/2358)	0.360
Arterial cord blood pH < 7.10	1.07% (29/2709)	1.57% (37/2358)	0.118
Arterial cord blood BE < −12	1.07% (29/2709)	1.15% (27/2358)	0.800
NICU hospitalization (all)	13.15% (357/2714)	13.14% (310/2359)	0.989
NICU hospitalization (childbirth related)	4.64% (126/2714)	3.01% (71/2359)	< 0.01
CPAP	1.22% (33/2714)	0.68% (16/2359)	0.051
Neonatal tracheal intubation	0.18% (5/2714)	0.08% (2/2359)	0.341
Hospitalization (days)	4.01 (±4.38)	3.71 (±3.93)	< 0.01
Hospitalization in NICU (days)	9.74 (±9.82)	9.54 (±8.35)	0.782
Neonatal composite outcome^a^	6.96% (189/2714)	5.34% (126/2359)	0.017

*LOA* left occiput anterior, *ROA* right occiput anterior, *BE* base excess, *CPAP* Continuous positive airway pressure, *CS* cesarean delivery, *NICU* neonatal intensive care unit

^a^Neonatal composite outcome consisting of one or more of the following outcomes: NICU hospitalization (childbirth related), low Apgar score at 5th minute (< 7), low cord blood pH (< 7.1), low cord blood base-excess (<−12), and neonatal tracheal intubation

### Cesarean section rate

**Fig. 1:**
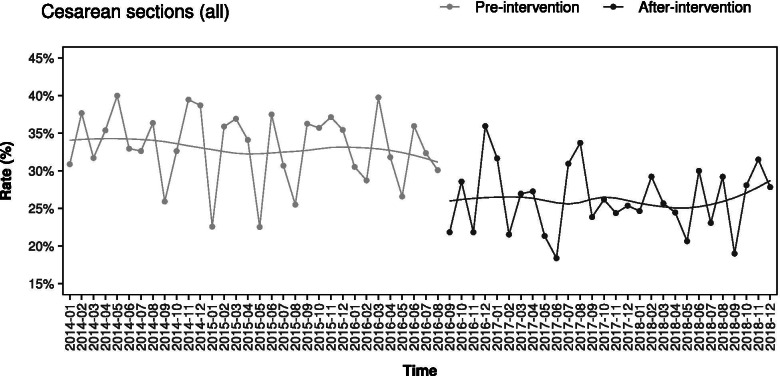
Overall cesarean sections rate

After starting interventions, the CS rate was dropped from 33.05 to 26.06% (*p* < 0.01) (Table 1 and Fig. 1). Figure 1 shows the monthly rate of CS. Already, from the beginning, there was a significant decrease in CS rate that was averagely maintained below the pre-intervention levels throughout the studied intervention period. The CS reduction was still statistically significant after correcting for possible confounding factors too (Table 4). An interrupted time series analysis was also performed. The time coefficient before intervention started was not significant (− 0.07, 95% CI -0.25/0.11, *p* = 0.441), showing no significant changes over time in the rate of CS in this period. On the contrary, the intervention coefficient was significant and negative after the intervention started (− 5.90, 95% CI -10.78/− 1.02, *p* < 0.05), indicating a substantial decrease in CS rate. Furthermore, the coefficient was not significant considering the period following the start of interventions (0.07, 95% CI -0.21/0.36, *p* = 0.616), showing a non-significant trend in CS rate.

**Table 4:** Univariate and multivariate logistic regression analysis

**CS**	OR (IC95%)	*p*	OR (IC95%)	*p*^†^
Intervention group	0.71 (0.63–0.81)	< 0.01	0.66 (0.57–0.76)	< 0.01
Maternal composite outcome^*^	OR (IC95%)	*p*	OR (IC95%)	*p*
Intervention group	1.12 (0.84–1.49)	0.448	–	–
**3rd-degree perineal tears**	OR (IC95%)	*p*	OR (IC95%)	*p*^‡^
Intervention group	2.12 (1.35–3.35)	< 0.01	2.36 (1.48–3.76)	< 0.01
Neonatal composite outcome^**^	OR (IC95%)	*p*	OR (IC95%)	*p*^§^
Intervention group	0.75 (0.6–0.95)	0.017	0.73 (0.57–0.93)	0.010

*BMI* body mass index, *CS* cesarean section, *DM* diabetes mellitus, *GDM* gestational diabetes mellitus, *PRHDs* pregnancy-related hypertensive disorders

^*^Maternal composite outcome consisting of one or more of the following outcomes: Perineal tears of 3rd or 4th-degree; post-partum hemorrhage; hysterectomy; fever; pulmonary embolism; hematoma; post-operative complications

^**^Neonatal composite outcome consisting of one or more of the following outcomes: NICU hospitalization (childbirth related), low Apgar score at 5th minute (< 7), low arterial cord blood pH (< 7.1), low arterial cord blood base-excess (<−12), and neonatal tracheal intubation

Multivariate logistic regression models:

^†^Corrected for maternal age, pre-pregnancy BMI, labor induction, labor augmentation, gestational age at delivery, multiple gestations, previous CS, pre-pregnancy DM, GDM, and PRHDs

^‡^Corrected for gestational age at delivery (weeks), neonatal weight > 4000 g, episiotomy, operative vaginal delivery

^§^Corrected for pre-pregnancy BMI (kg/m^2^), labor induction, gestational age at delivery (weeks), previous CS, DM, GDM, PRHDs, neonatal weight > 4000 g, operative vaginal delivery

The rate of elective CS (primary CS) was only slightly decreased, dropping from 13.59 to 11.96% (*p* = 0.086), while the rate of CS during labor (secondary CS) was significantly decreased from 19.46 to 14.11% (*p* < 0.01). Besides, the emergency CS remained stable after starting the intervention (Table 1).

Table 2B shows the CS prevalence subdivided according to Robson classes. In Robson class 1, 2a, 4a, 5a, and 8 there was a significant decrease in CS rate after starting the intervention.

### Operative vaginal delivery and other maternal morbidities

**Fig. 2:**
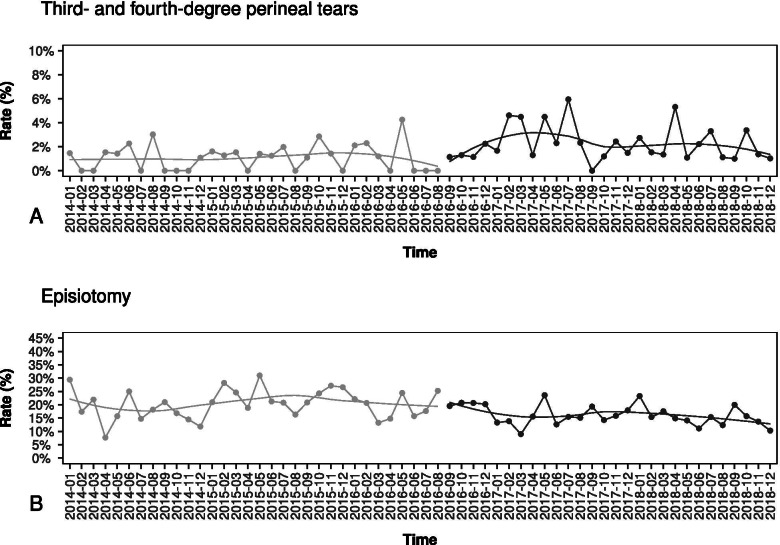
Overall third- and fourth-degree perineal tears and episiotomy rate. Panel **A**: Third- and fourth-degree perineal tears; Panel **B**: Episiotomy rate

There was a nonsignificant decrease in the rate of vaginal-operative births (Table 1), despite the reduction of CS rate after starting the intervention period. Besides, there was a significant reduction in episiotomy rate and in maternal hospitalization times (both considering all women and only CS subgroup) (Table 1). The prevalence of the maternal composite outcome and most related single components considered were not statistically different before and after the intervention (Table 1). There was a non-significant reduction in postpartum hemorrhage after the introduction of the intervention, and no neonatal mortality events were registered. However, accompanied by a decrease in episiotomy rate, high-degree perineal tears increased after the start of the intervention, mainly due to an increase in 3rd-degree tears (Table 1 and Fig. 2, panel A-B). This increase was also significant after correcting for possible confounding factors (Table 4).

### Neonatal morbidity

The composite negative neonatal outcome was statistically reduced after starting intervention (from 6.96 to 5.34%, *p* = 0.017) (Table 3). This difference was also statistically significant after correction for possible confounding factors in multivariate logistic regression analysis (Table 4). Specifically, there was a reduction in low Apgar score (< 7) at 5 min, use of CPAP, and NICU hospitalization for childbirth related complications (Table 3). Also, neonatal hospitalization length was significantly reduced (*p* < 0.01).

## Discussion

Our data show how adoption of a few simple interventions can rapidly and significantly reduce the local rate of cesarean section deliveries. Results were accompanied by a positive effect on composite neonatal outcome, however, with an increase in the rate of third-degree perineal tears.

The background of our study initiated for reducing the cesarean section rate was the preliminary analysis of the data of the previous year provided by the institute for quality assurance and transparency in health care (Institut für Qualitätssicherung und Transparenz im Gesundheitswesen, IQTIG). This agency supplies a comparison of local with regional data concerning cesarean section rate and other obstetrical outcomes of the pregnancy and delivery. According to these data, CS rate in year 2015 at our institution accounted for 32.46% of all deliveries, while nation-wide CS rate amounted to 31.42% [15]. In the same year, regional (North-Rhein-Westphalia, NRW) cesarean section rate accounted for 32.47%, in line with our data. However, NRW hospitals reported, as expected, a wide distribution of the CS rate with a minimum of 12.49% and a maximum of 58.02% [16]. According to these data, we supposed that there would be potential margins for reducing the cesarean section rate in our hospital.

For this scope, we chose to perform an intervention study. During a preliminary phase, a proposal for interventions at different levels were made by a dedicated task force using a Delphi-oriented approach. This approach allowed reaching a wider consensus on each task needed to be addressed. Also, a down-up oriented changing process of organizational and personal workflows allowed a deeper motivation and acceptance of the interventions proposed [17].

Among strategies for improving natural childbirth, those proposed at an organizational level were the easiest to be started. The decision to form a task force of dedicated senior gynecologists, responsible for the delivery room seems to us of paramount importance. One of the initiators of the study (AF), was a new senior gynecologist with perinatal competences having joined the staff 2 months prior to the start of the study. The rotation in back-up role during the last year of the study avoided the results of the study from being dependent upon a single researcher. Furthermore, these two senior gynecologists responsible for the delivery room were also responsible for the prenatal interview of women desiring to give birth at our institution. This included a counseling concerning delivery mode and discussion of expected issues. This pre-admission interview allowed uniformity of the counseling and had the great advantage of guaranteeing proper continuity in women’s care and enforcing the patient-gynecologist relationship. Of note, the antecedent hospital organization contemplated a daily rotation of all senior gynecologists to be responsible both for the delivery room during the day shift as well as for the ante-natal interviews.

Another task, addressed to health-care professionals, was the improvement of the competence of the obstetrical personnel, both practical and theoretical, introducing a monthly internal educational training, and morbidity & mortality conferences. Coupling theoretical knowledge with practical training concerning clinical cases became ongoing permanent educational training to reduce unmotivated fears and support an evidence-based obstetrical culture. The presence of both the obstetrical and midwifery teams facilitated steady feedback and, finally, a better cooperation between the two teams.

Nonetheless, results of our interventions showed a significant reduction of CS rate following the introduction of the different interventions. The sub-group analysis according to the Robson classification, showed a significant reduction of CS performed was registered in nulliparous women with singleton cephalic pregnancies at term, both during spontaneous or induced labor (respectively Robson 1 and 2a). A significant reduction was also achieved in another large group of patients, the multiparous women with singleton cephalic pregnancies at term, both without or with a previous CS scar, even though in this latter group, only during spontaneous labor (respectively Robson 4a and 5a). A numerical less relevant reduction in CS rate was achieved in the group of women with multiple pregnancies (Robson 8). The cumulative analysis of CS performed during labor (secondary CS) showed a significant reduction in CS rate, while elective CS (primary CS) showed no reduction. These results reflect the difficulty of addressing the group of CS due to absolute fetal or maternal indication, as well as those with a relative medical contraindication to vaginal birth or due to maternal choice. Interestingly, no increase was observed in operative vaginal delivery rate.

A change in the organizational structure played, apparently, a relevant role, being linked to the provision of a consistent and skilled pre-labor counseling and labor management. The presence of a specialist in perinatology and the provision of further interventions focused on increasing practical and theoretical competence of healthcare professionals through continual educational training provided a stabilization of results achieved in the first period [18]. Interestingly, CS rate dropped rapidly and remained at a low level, also after the senior gynecologist responsible for the delivery room during the day changed in the last year.

Concomitant to the CS rate reduction, a significant increase in 3rd-degree perineal lacerations was observed. Of note, this increase concerned only the 3rd-degree perinatal tears, while the 4th-degree tears were unchanged. This increase could be explained through the higher number of vaginal deliveries, including those ones at higher risk that would undergo a CS before the intervention started. Furthermore, besides this increase in high-degree perineal laceration, we observed a substantial decrease in episiotomy rate, which could also be interpreted as a not previewed change in obstetrical attitude toward prophylactic episiotomy. Of note, a tendency toward a reduction of 3rd-degree perineal tears after the initial increase was observed, probably because of a further ongoing adaptation in our obstetrical practice to prevent high-degree perineal tears during vaginal birth (see Fig. 2, right hand of panel B). Furthermore, we did not observe any increase in further adverse maternal outcomes and in the composite negative outcome during the overall study-period. On the contrary, we observed a significant amelioration in several neonatal outcomes, including the 5th minute Apgar score < 7, the childbirth related NICU hospitalization, the length of hospital stays, as well as the composite negative neonatal outcome including all neonatal complications evaluated.

The feasibility of a safe reduction of cesarean section rate has been described by several studies. According to a meta-analysis performed by Chaillet e Dumont in 2007, a significant reduction of cesarean section rate was found by random meta-analysis of 10 included studies evaluating the effectiveness of different strategies on cesarean rate reduction [19]. Pooled data were also separately evaluated for audit and feedback, quality improvement-based management of labor and multifaceted strategies, showing a positive effect on cesarian rate for all types of interventions. Importantly, no significant differences were found for perinatal and neonatal mortality and morbidity concerning the mode of delivery among included studies [19].

Addressing specific factors contributing to the rise in CS rate could be particularly challenging, as causes are generally complex and multifactorial [20]. Changes in clinical features of pregnant women and newborns, like the increasing percentage of nulliparous women or the increasing maternal age and presence of comorbidities during pregnancy, play a relevant role and should be addressed, even though they are difficult to be targeted [21–23]. Beside these factors, there are non-clinical factors concerning the organization of health-care services and the skills and motivation of both health-care providers and pregnant women, that can be more easily addressed at a local level [24, 25]. According to our experience, the intervention that most influenced the decrease of CS rate was probably the introduction of a dedicated team of obstetricians for delivery room and antenatal counseling. Indeed, we were able to document a drop in CS rate soon after the initiation of the study. This intervention helped the obstetrical and midwifery team to work together in a more homogeneous way, avoiding changes in the management due to the frequent rotations of the obstetricians responsible for the delivery room and antenatal counseling we had before the study started. The other interventions introduced were also important, probably helping to strengthen results by promoting natural birth through an evidence-based obstetrical culture.

A recent Cochrane review was performed to evaluate the effectiveness and safety of non-clinical interventions intended to reduce unnecessary caesarean section in agreement with the above-cited WHO classification [26]. According to the analysis of the 29 studies included, interventions targeted to healthcare professionals showed a range of moderate to high grade of evidence for a safe reduction of CS rate. These interventions included the implementation of guidelines combined with mandatory second opinion, the implementation of guidelines combined with audit and feedback, and physician education by local opinion leader [27–32]. Unclear to low-certainty evidence has been reported regarding interventions targeted at women [33–35]. Furthermore, concerning the healthcare facilities, even though financial interventions targeted at healthcare professionals did not show substantial effects, a different staffing model of delivery care seems to be able to provide a reduction in CS rate [36, 37]. A collaborative midwifery-obstetric care in which the obstetrician’s presence during labor and delivery and whose focus was solely on the labor and delivery unit without other competing clinical duties, was shown to reduce CS rate [38, 39].

Recent research has further focused on a better definition and evaluation of specific measurable criteria related to the efforts of a CS reduction. Regardless of the well-known overall CS rate, the effect of applied interventions could be evaluated in different categories of cesarean section according to the Robson’s 10-group CS classification [40]. This type of subdivision proposed has the advantage of allowing a standardized, easy to apply and more meaningful clinical classification of the different types of cesarean sections depending on the CS indication and type of pregnancy. Beside this classification, it has been recently proposed to focus the evaluation of cesarean section rate in nulliparous term singleton vertex pregnancies, using the Nulliparous Term Singleton Vertex Cesarean Birth (NTSV CB) measure. This tool has the potential to provide stable and powerful data, including a greater sample of pregnant women. Furthermore, by limiting the group evaluated to these women, generally no further risk adjustment is thought to be needed [41]. Other metric tools for quality improvement evaluation have been proposed, one of them by the Society of Maternal–Fetal Medicine Cesarean Birth Metrics [42]. This tool considers all term, singleton, and cephalic presentations, has shown to have the advantage of providing a larger and more stable comparison tool between hospitals compared to the nulliparous term singleton vertex cesarean birth tool [43].

### Limits of the study

In general, a relevant limitation of pre-post intervention studies conducted using a retrospective control group, is the lack of comparable controls. To limit this drawback, a multivariate analysis controlling for potential confounding factors was performed. According to the data there was no significant difference in the baseline characteristics among the study group and the historical control group. Nevertheless, we cannot exclude that some results are linked to other changes in obstetrical practice not included in the interventions taken in place. This could be the case, at least partially, of the significant increase in 3rd-degree perineal tears, even after multivariate analysis correcting for episiotomy rate reduction.

Furthermore, due to the multiplicity of the interventions introduced there is no possibility for a direct control on the effect produced by each one. However, scope of the study was not to evaluate the effect of a single intervention, but to analyze the cumulative effect of a series of interventions potentially influencing this outcome, as does indeed occur in real clinical practice.

## Conclusions

Our study shows that a significant reduction in the CS rate can be achieved through few and simple interventions. Besides a potential increase of high-degree perineal tears, interventions were accompanied by an amelioration in the composite neonatal outcome. Thus, beyond general recommendations, this study shows that each single medical institution should be encouraged to develop specific strategies targeted to support natural childbirth at a local level.

## Supplementary Information

**Additional file 1: Supplemental Table 1.** The Robson Classification (According to “Robson Classification: Implementation Manual”. Geneva: WHO; 2017. Licence: CCBY-NC-SA 3.0 IGO).

## Data Availability

The data that support the findings of this study are available from the authors upon reasonable request and with permission of the Internal Review Board.
