# Supplementary material for: Composite non-clinical interventions for a safe cesarean section rate reduction: results of a pre-post interventional study
Source: BMC Pregnancy Childbirth. 2021 Nov 19;21:783. doi: 10.1186/s12884-021-04245-y (PMC8603588; doi:10.1186/s12884-021-04245-y)
Supplement: Supplementary file 1 — Additional file 1: Supplemental Table 1. The Robson Classification (According to “Robson Classification: Implementation Manual”. Geneva: WHO; 2017. Licence: CCBY-NC-SA 3.0 IGO). [file 12884_2021_4245_MOESM1_ESM.docx]

**Supplemental Table 1**- The Robson Classification (According to “Robson Classification: Implementation Manual”. Geneva: WHO; 2017. Licence: CCBY-NC-SA 3.0 IGO.)

| Group | Obstetric population | Label |
| --- | --- | --- |
| 1 | Nulliparous women with a single cephalic pregnancy, ≥37 weeks gestation in spontaneous labour | Nulliparous single term cephalic pregnancy in spontaneous labor |
| 2 | Nulliparous women with a single cephalic pregnancy, ≥37 weeks gestation who had labour induced or were delivered by CS before labour |  |
| 2a | Labour induced | Nulliparous single term cephalic pregnancy (labor induced) |
| 2b | Pre-labour CS | Nulliparous single term cephalic pregnancy (pre-labor CS) |
| 3 | Multiparous women without a previous CS, with a single cephalic pregnancy, ≥37 weeks gestation in spontaneous labour | Multiparous (no previous CS) single term cephalic pregnancy in spontaneous labor |
| 4 | Multiparous women without a previous CS, with a single cephalic pregnancy, ≥37 weeks gestation who had labourinduced or were delivered by CS before labour |  |
| 4a | Labour induced | Multiparous (no previous CS) single term cephalic pregnancy (labor induced) |
| 4b | Pre-labour CS | Multiparous (no previous CS) single term cephalic pregnancy (pre-labor CS) |
| 5 | All multiparous women with at least one previous CS, with a single cephalic pregnancy, ≥37 weeks gestation |  |
| 5.1 | With one previous CS | Multiparous (one previous CS) single term cephalic pregnancy |
| 5.2 | With two or more previous CSs | Multiparous (≥2 previous CS) single term cephalic pregnancy |
| 6 | All nulliparous women with a single breech pregnancy | Nulliparous single breech pregnancy |
| 7 | All multiparous women with a single breech pregnancy including women with previous CS(s) | Multiparous single breech pregnancy (included previous CS) |
| 8 | All women with multiple pregnancies including women with previous CS(s) | Multiple pregnancies (included previous CS) |
| 9 | All women with a single pregnancy with a transverse or oblique lie, including women with previous CS(s) | All women single pregnancy with transverse or oblique fetal lie (included previous CS) |
| 10 | All women with a single cephalic pregnancy < 37 weeks gestation, including women with previous CS(s) | All women with single pre-term cephalic pregnancy (included previous CS) |
